# Muscle Activation During ACL Injury Risk Movements in Young Female Athletes: A Narrative Review

**DOI:** 10.3389/fphys.2018.00445

**Published:** 2018-05-15

**Authors:** Jesper Bencke, Per Aagaard, Mette K. Zebis

**Affiliations:** ^1^Human Movement Analysis Laboratory Section 247, Department of Orthopedic Surgery Section 333, Hvidovre Hospital, Copenhagen University Hospital at Amager-Hvidovre, Copenhagen, Denmark; ^2^Department of Sports Science and Clinical Biomechanics, Research Unit for Muscle Physiology and Biomechanics, University of Southern Denmark, Odense, Denmark; ^3^Department of Physiotherapy and Occupational Therapy, Faculty of Health and Technology, Metropolitan University College, Copenhagen, Denmark

**Keywords:** adolescent, female, athlete, ACL, injury risk, muscle activation, hamstrings, injury prevention

## Abstract

Young, adolescent female athletes are at particular high risk of sustaining a non-contact anterior cruciate ligament (ACL) injury during sport. Through the last decades much attention has been directed toward various anatomical and biomechanical risk factors for non-contact ACL injury, and important information have been retrieved about the influence of external loading factors on ACL injury risk during given sports-specific movements. However, much less attention has been given to the aspect of neuromuscular control during such movements and only sparse knowledge exists on the specific muscle activation patterns involved during specific risk conditions. Therefore, the aim of this narrative review was (1) to describe anatomical aspects, strength aspects and biomechanical aspects relevant for the understanding of ACL non-contact injury mechanisms in young female athletes, and (2) to review the existing literature on lower limb muscle activation in relation to risk of non-contact ACL-injury and prevention of ACL injury in young female athletes. Studies investigating muscle activity patterns associated with sports-specific risk situations were identified, comprising cohort studies, intervention studies and prospective studies. Based on the retrieved studies, clear gender-specific differences in muscle activation and coordination were identified demonstrating elevated quadriceps activity and reduced hamstring activity in young female athletes compared to their male counterparts, and suggesting young female athletes to be at elevated risk of non-contact ACL injury. Only few studies (*n* = 6) examined the effect of preventive exercise-based intervention protocols on lower limb muscle activation during sports-specific movements. A general trend toward enhanced hamstring activation was observed during selected injury risk situations (e.g., sidecutting and drop landings). Only a single study examined the association between muscle activation deficits and ACL injury risk, reporting that low medial hamstring activation and high vastus lateralis activation prior to landing was associated with an elevated incidence of ACL-injury. A majority of studies were performed in adult female athletes. The striking paucity of studies in adolescent female athletes emphasizes the need for increased research activities to examine of lower limb muscle activity in relation to non-contact ACL injury in this high-risk athlete population.

## Introduction

Acute knee injury, especially injury to the anterior cruciate ligament (ACL), represents a serious problem in ball sports and racket sports that involve abrupt changes of direction, i.e., landing, turning, and sidecutting (Myklebust et al., 1997; Faude et al., 2006; Beynnon et al., 2014; Pasanen et al., 2017). Most of these injuries occur in non-contact conditions, and in contrast to acute contact injuries the risk of sustaining non-contact injuries appears to be related to neuromuscular factors influencing the biomechanical loading of the knee (Hewett et al., 2005a), as these factors in turn affects the magnitude and timing of muscular force production that can serve to stabilize the knee (Hewett et al., 2005b; Zebis et al., 2009). Adolescent female athletes appear to be at particular high risk of sustaining non-contact ACL injury (Renstrom et al., 2008; Lind et al., 2009). Across sports, the overall incidence of a first-time non-contact ACL rupture in female high school and college athletes have been reported to be as high as 0.112 per 1000 athlete exposures compared with 0.063 per 1000 athlete exposures in males (Beynnon et al., 2014), and ACL injury incidence rates for women seems to peak during adolescence (age 14–18 years) with 227.6 (per 100,000 person years) compared to 113.2 (per 100,000 person years) for the following age group (age 19–25 years) (Sanders et al., 2016). After ACL rupture, the consequences for the individual may be severe, both in short term when the activity in the given sport is paused and potentially have to be terminated, but also in the long term reflected as an increased risk of early onset of osteoarthritis (Molloy and Molloy, 2011) and long-term quality of life (QOL) impairment (Filbay et al., 2017). ACL injury prevention therefore seems of outmost importance in young female athletes, which prompts for a strong need for optimized prophylactic training regimes targeting this specific age- and sex- group.

In order to design effective injury prevention programs, influential risk factors must be identified (van Mechelen et al., 1992). Describing the anatomy of the ACL and the internal lever arms of relevant muscles around the knee joint will yield relevant information about movements in all three planes that impose stress forces on the ACL, and enable to identify which muscles could act as antagonist or synergists to the ACL in different movements and joint positions. Also, gender and age dependent development of muscle strength in these muscle groups would aid to understand the challenges facing young female athletes in sports-specific injury risk situations. Furthermore, analyzing the magnitude and timing of biomechanical loading on the knee joint and the ACL during such risk situations and their relationship to age or gender would also help to improve our understanding of which muscle groups that need to be strong and/or highly active during specific injury risk situation in order to reduce the risk of non-contact ACL injury in young female athletes. A large number of reviews have examined selected anatomical, strength-related and biomechanical aspects (currently 32 review papers can be identified on PubMed using the search terms: ACL AND “injury risk” AND (anatomical OR biomechanical OR physiological) AND (Review[ptyp])) whereas only few review studies have investigated how specific patterns of muscle activation may influence the risk of ACL injury (three review papers retrieved, when adding “EMG” to the previous search string). Even less is known about the effect of specific training intervention on adapting muscle activation patterns that are more in favor of protecting the ACL from non-contact injury in young female athletes.

Thus, the purpose of this narrative review was (1) to describe anatomical aspects, strength aspects and biomechanical aspects relevant for the understanding of ACL non-contact injury mechanisms in young female athletes, and (2) to review the existing literature on lower limb muscle activation in relation to risk of non-contact ACL-injury and prevention of ACL injury in young female athletes. The findings and conclusions of this review are expected to help health care professionals including physiotherapists and exercise physiologists, coaches and physical trainers to design and implement more efficient and varied exercise programs for ACL injury prevention in young female athletes.

### Anatomical Aspects

From cadaveric studies it has been shown that the anatomical function of the ACL is to add to the passive stability of the knee joint in all three planes (Markolf et al., 1995). In the sagittal plane forward translation of the tibia is restrained by the ACL, and the resulting anterior forward pull on the ACL decreases with increased knee flexion (Markolf et al., 1995; Fleming et al., 2001), while active quadriceps contraction force is a major contributor to this anterior shear force (Renstrom et al., 1986; Draganich and Vahey, 1990; More et al., 1993; Durselen et al., 1995). Due to the patella ligament angular attachment to the tibia, the contribution of quadriceps muscle contraction force to anterior shear force is most pronounced at more extended knee joint angles of 0–35 degrees of flexion (Beynnon et al., 1995; Durselen et al., 1995; Fleming et al., 2001). Thus these data show, from a biomechanical-anatomical perspective, that large force generation in the knee extensors concurrent with more extended knee joint angles during forceful landing or sidecutting increase the magnitude of loading (strain) in the ACL in the sagittal plane and hence contribute to an increased risk of ACL injury.

In the frontal plane, the ACL restrains knee abduction and adduction movement, as seen by increased ACL strain when loading the knee in either valgus or varus direction in combination with anterior tibial shear force production (Markolf et al., 1995). In the transverse plane internal rotation of the tibia has been shown to add to the loading of the ACL (Durselen et al., 1995; Markolf et al., 1995; Fleming et al., 2001), while external rotation in combination with valgus may cause similar effects when the ACL is restrained by the medial-anterior aspect of the lateral condyle (Ebstrup and Bojsen-Moller, 2000; Olsen et al., 2004). Anatomical and computer modeling studies have demonstrated that the hamstring muscles play an important role as ACL synergists by providing posterior tibial stress forces that countermeasure anteriorly directed stress forces in the knee joint (Renstrom et al., 1986; Draganich and Vahey, 1990; Pandy and Shelburne, 1997; Li et al., 1999; MacWilliams et al., 1999). The importance of the hamstring muscles for providing dynamic knee joint stability is further elaborated by the fact that ACL serves a neural function as an important site of proprioceptive feed-back in the control of muscles around the knee joint. Dyhre-Poulsen and Krogsgaard (2000) demonstrated that reflex pathways are present in humans, where long-latency (∼120 ms) reflex responses were recorded in the hamstring muscles following electrical intra-articular stimulation of the ACL (Dyhre-Poulsen and Krogsgaard, 2000; Krogsgaard et al., 2002).

### Muscle Strength Aspects

Gender differences in maximal lower limb muscle strength are strongly manifested in adult athletic populations, however these differences are not apparent among immature children (McKay et al., 2017). In general, females are not found to improve maximal lower limb strength expressed relative to body mass during maturation (DiStefano et al., 2015). For thigh muscle strength, boys appear to increase strength more than girls during maturation, and in addition strength development in the hamstrings compared to the quadriceps seems to be less favorable developing in girls, leading to decreased H/Q strength ratios in post-pubertal girls (Ahmad et al., 2006). These changes in mechanical muscle properties may put young female athletes at elevated risk of ACL injury compared to their male counterparts.

### Biomechanical Aspects

A viable biomechanical approach to investigate the mechanistic causes of a non-contact ACL injury could be to analyze the specific sports movements recognized as high risk situations for sustaining ACL injury, in order to examine various biomechanical factors that may result in an increased ACL strain during specific types of movement. Increased insight into gender specific differences in biomechanical loading and/or neuromuscular activation patterns during such sports specific risk situations may further elucidate why young female athletes appear more susceptible to non-contact ACL injury (Renstrom et al., 2008), and more importantly provide guidance toward more effective countermeasures to prevent such injuries.

Landing from a jump, run-to-stop and sidestep cutting maneuvers are frequently occurring situations that all have been associated with increased risk of ACL injury in a variety of sports (Myklebust et al., 1997; Faude et al., 2006; Krosshaug et al., 2007; Beynnon et al., 2014; Pasanen et al., 2017), and advanced biomechanical analyses of individual injury cases have shown that ACL rupture typically occurs within the first 50 ms after initial ground contact (Krosshaug et al., 2007). In order to identify which biomechanical risk factors that may be represented in various risk movement tasks, previous experiments primarily have been carried out in biomechanical laboratories and often using combined recordings of 3D kinematics/kinetics, ground reaction forces and electromyography (discussed below in the section on muscle activation).

Based on the available data, female athletes tend to demonstrate more extended knee and hip joint angles than their male counterparts during vertical and horizontal landings as well as in lateral sidecutting maneuvers (Malinzak et al., 2001; Lephart et al., 2002; Fagenbaum and Darling, 2003; McLean et al., 2004, 2005; Ford et al., 2005; Chappell et al., 2007; Schmitz et al., 2007). This gender difference seems to emerge during maturation, where post-pubertal girls are noted to land with more extended knee joint angles compared to pre-pubertal girls (DiStefano et al., 2015). Use of extended knee joint positions during landing has been associated with elevated anterior shear forces in the knee joint and hence presumably elevated ACL strain in female study participants (Chappell et al., 2002; Shultz et al., 2009; Tsai and Powers, 2013; Tsai et al., 2017). Likewise, computer model analysis of landings have uniformly indicated that landing/cutting at more extended hip and knee joint angles are predictive of elevated anterior tibial shear forces most likely resulting in increased strain in the ACL (Sell et al., 2007; Southard et al., 2012; Tsai and Powers, 2013; Tsai et al., 2017). Conversely, landing or sidecutting with more flexed knee joint angles has been reported to reduce the magnitude of anteriorly directed strain in the ACL, but also to facilitate the hamstrings to act as ACL synergists as demonstrated in cadaveric studies and using computer modeling (Li et al., 1999; Kernozek and Ragan, 2008; Southard et al., 2012). A recent study stressed the importance of avoiding extended knee joint angle during foot strike by prospectively investigating drop jump landing and showing that landing with more extended knee joint angles and increased ground reaction forces were associated with subsequent ACL injury in a cohort of young female basketball and floorball athletes (Leppanen et al., 2017).

**FIGURE 1:**
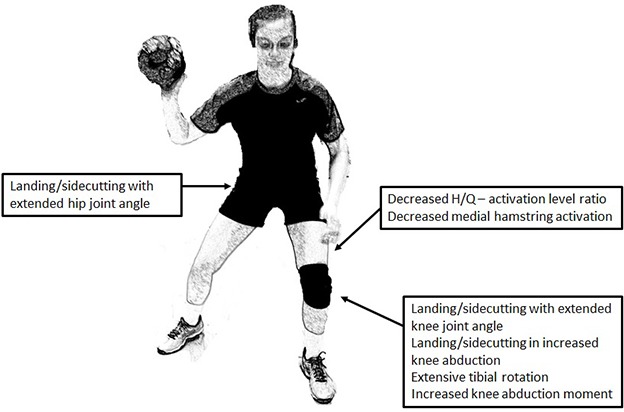
Risk factors for non-contact ACL injury in young female athletes.

Besides causing unfavorable strain effects in the ACL in the sagittal (anterior–posterior) plane, landings performed with more extended knee joint angles may also affect knee joint loading in other planes. Thus, landing with a more extended knee increases the magnitude of vertical ground reaction impact forces, causing increased compression of the knee joint, which due to the posterior slope angle of the tibial plateau may induce internal tibial rotation that leads to increased strain in the ACL (Torzilli et al., 1994; Kernozek and Ragan, 2008). In addition, performing landing with more extended knee joint positions have been associated with elevated external knee abduction moments in the frontal plane (Pollard et al., 2010), and gender differences in frontal plane kinematic and kinetic differences have often been observed during landing or sidecutting (Ford et al., 2003, 2005; McLean et al., 2004, 2005) (**Figure 1**). These observations have led to the hypotheses that frontal plane biomechanics are important to consider as separate and significant risk factors for non-contact ACL injury, especially in female athletes (Quatman and Hewett, 2009).

In strong support of this notion, using a prospective study design Hewett et al. (2005a) observed an increased risk of subsequent ACL injury in young female athletes who demonstrated increased external knee abduction moment and increased valgus angle at initial contact during drop jump testing. Further, although no differences in knee joint flexion were noted at initial contact, the degree of maximal knee flexion (i.e., functional range of motion) during the landing was reduced in athletes later sustaining ACL injury (Hewett et al., 2005a). On the other hand, prospective data obtained in elite female football and handball players have not been able to verify this relationship to ACL injury incidence despite also using drop jump testing (Krosshaug et al., 2016). Studies examining sidestep cutting maneuvers generally have reported elevated external moments around the knee joint compared to studies examining drop jumps, while also displaying coinciding peaks of external knee flexion moments, knee abduction moments and tibia rotational moments within the first 50 ms after landing in female athletes (Bencke et al., 2013; Kristianslund and Krosshaug, 2013). It is likely that a combination of all of these potentially ACL-stressing joint moments may increase the risk of sustaining a non-contact ACL-injury.

While the above biomechanical risk factors can be considered general across all age groups, specific gender differences do not seem to appear until after puberty where distinct sex differences in landing biomechanics become noticeable (Hewett et al., 2004; Quatman et al., 2006). The resulting modifications in knee loading patterns may be more challenging for young adolescent female athletes, given that the concurrent deficit in hamstring strength development relative to the quadriceps (Ahmad et al., 2006) may reduce the ability to generate synergistic joint forces that are protective to the ACL. However, much less data exist on the influence of muscle activation patterns on the risk of ACL injury, especially in young female athletes despite that this population is at particular risk of sustaining such type of injury.

### Muscle Activation

The present narrative review intended to examine the significance of muscle activation on the risk of non-contact ACL injury, and to investigate the effect of preventive measures on a reprogramming in muscle activation patterns in young female athletes. Retrieved study papers were divided into three categories: (i) studies investigating muscle activation patterns related to biomechanical risk factors or gender differences in muscle activation patterns (**Table 1**); (ii) studies directly associating muscle activation patterns to the risk of sustaining non-contact ACL injury using prospective designs (**Table 2**), and (iii) studies investigating the effect of preventive exercise training on the reprogramming in muscle activation patterns (**Table 3**).

**Table 1:** Studies relating neuromuscular activation to biomechanical risk factors or gender comparisons.

Study	Female/male	Age [years (SD)]	Outcome parameters	Results
Beaulieu et al., 2009	*N* = 15/15	F: 21.1 (3.6) M: 22.9 (3.7)	Sidecut 3D kinematics and quadriceps, hamstring and gastrocnemius EMG. Gender differences	Females more gastrocnemius activity than males, and a more VL-to-VM dominated activity than males
Bencke and Zebis, 2011	*N* = 12/12	F: 22.7 (3.1) M: 23.1 (3.4)	Sidecut preactivity EMG of quadriceps and hamstring, and pre-activity H/Q-ratio	Females showing less hamstring preactivity and smaller H/Q-ratio
Chappell et al., 2007	*N* = 19/17	F: 22.3 (2.2) M: 22.6 (2.2)	Stop jump 3D biomechanics and averaged and normalized quadriceps and hamstring EMG	Females more quadriceps activation than males, and more hamstring activation before landing
Colby et al., 2000	*N* = 6/9	F+M: 22.2 (1.7)	Sidecut and landing kinematics and quadriceps and hamstring EMG	High quadriceps activity and low hamstring activity before ground contact
Ebben et al., 2010	*N* = 12/12	F: 19.9 (0.9) M: 21.0 (1.2)	Sidecut and drop landing quadriceps and hamstring EMG	Females showing less hamstring activity than males after contact, and lower EMG H/Q-ratio
Fagenbaum and Darling, 2003	*N* = 8/6	University athletes	Jump landing knee kinematics and quadriceps and hamstring EMG	No gender differences in EMG activity
Hanson et al., 2008	*N* = 20/20	F: 19.8 (1.1) M: 19.4 (1.4)	Sidecut quadriceps and hamstring and hip EMG	Females showing greater pre-activity VL and gluteus medialis activation and lower H/Q-ratio than males
Landry et al., 2007	*N* = 21/21	F: 16.7 (1.0) M: 17.0 (0.6)	Sidecut 3D biomechanics and EMG of quadriceps, hamstring and gastrocnemius	Females demonstrating higher quadriceps and gastrocnemius activity in stance
Malinzak et al., 2001	*N* = 9/11	F: 24.6 (1.0) M: 24.5 (2.5)	Running, cross-cutting, and sidecutting 3D biomechanics and quadriceps and hamstring EMG	Females tending to have elevated quadriceps and reduced hamstring activity during stance phase in all movement task examined
Myer et al., 2005	*N* = 10/10	F: 22.3 (3.7) M: 25.5 (2.7)	Slow lateral knee flexion and extension measuring VL and VM activity	Females having lower VM/VL ratio
Nagano et al., 2007	*N* = 19/18	F: 19.4 (0.9) M: 19.8 (4.6)	Single leg drop landing 3D biomechanics and quad-riceps and hamstring EMG	Pre activity EMG H/Q-ratio was lower in females compared to males
Pollard et al., 2010	*N* = 58/0	13.5 (range: 11–20)	Drop landing 3D biomechanics. Quadriceps and hamstring EMG	Female subjects with more extended knee joint angle at landing impact showing increased quadriceps EMG
Russell et al., 2007	*N* = 28/27	Children: 9.5 (0.9) Adults: 23.9 (2.8)	Drop landing 3D biomechanics and H/Q-activation ratio between children and adults	Children showing smaller EMG H/Q-ratios compared to adults
Sell et al., 2007	*N* = 17/19	F: 16.1 (1.3) M: 16.3 (1.5)	Stop jump 3D biomechanics and VL and ST EMG	VL activity related to anterior shear force
Shultz et al., 2009	*N* = 39/39	F: 22.2 (2.9) M: 22.6 (2.6)	Drop Jump 3D biomechanics and quadriceps and hamstring EMG	Females having more quadriceps and hamstring activity than males. Greater peak quadriceps activity in subjects with high anterior shear forces, regardless of sex. Low strength was moderately related to high quadriceps activity in females.
Sigward and Powers, 2006a	*N* = 30/0	F: 15.4 (1.0)	Sidecutting 3D biomechanics and quadriceps and Hamstring EMG differences between experienced and novice athletes	Novice athletes showed greater antagonist-agonist muscle co-activation at the knee
Sigward and Powers, 2006b	*N* = 15/15	F: 19.4 (1.5) M: 19.6 (1.9)	Sidecutting 3D biomechanics and quadriceps and hamstring EMG	Females displayed greater average quadriceps EMG
Zazulak et al., 2005	*N* = 13/9	Division I athletes (United States)	Drop landing hip and thigh EMG	Females showed less gluteus max activity after landing and more quadriceps pre activity

*Only neuromuscular study data are summarized.*

*VM, m.vastus medialis; VL, m.vastus lateralis; H/Q-ratio, Hamstring-to Quadriceps ratio.*

**Table 2:** Studies directly associating muscle activation patterns to risk of ACL injury using prospective study designs.

Study	Cohort/follow-up	Age (SD)	Outcome parameters	Results
Zebis et al., 2009	Fifty five female non-injured athletes were followed for two seasons	24 (5) years	Sidecutting EMG of quadriceps and hamstrings	Five ACL injuries registered at follow-up. At baseline, the injured group showed less medial hamstring activity and greater VL activity, along with an elevated VL-ST EMG difference

*Only muscle activation study data are summarized.*

**Table 3:** Studies examining effect of training on muscle activation in female athletes.

Study	Intervention/control	Age [years (SD)]	Outcome parameters	Results
Lephart et al., 2005	Plyometric (*n* = 14)/resistance training (*n* = 13)	I: 14.5 (1.3) C: 14.2 (1.3)	Jump-landing 3D biomechanics and hip and thigh EMG	Both training groups increased gluteus medius activity before and during landing
Letafatkar et al., 2015	Pertubation training (*n* = 15)/control (*n* = 14)	I and C: 24.3 (3.5)	Single leg drop landing EMG of quadriceps and hamstrings	Increased level of co-contraction between quadriceps and hamstrings in the intervention group after intervention
Nagano et al., 2011	Jump and balance training (*n* = 8)/no control group	I: 19.4 (0.7)	Single leg drop landing 3D biomechanics and quadriceps and hamstrings EMG	Intervention increased pre activity hamstring activation, but not H/Q-ratio
Wilderman et al., 2009	Agility training (*n* = 15)/control group (*n* = 15)	I: 21.1 (3.6) C: 21.1 (1.8)	Sidecutting 3D kinematics and quadriceps and hamstrings EMG	Intervention group increased medial hamstring activation during ground contact
Zebis et al., 2008	Balance+jump+landing (*n* = 20), compare control season vs. intervention season	I: 26 (3)	Sidecutting EMG of hip thigh and shank muscles	No change during control season but reduction in gluteus medialis pre activity and increase in medial hamstring activity following the intervention protocol
Zebis et al., 2016	Balance+jump+landing (*n* = 20)/control (*n* = 20)	I: 15.9 (0.4) C: 15.6 (0.5)	Sidecutting 3D biomechanics and EMG of quadriceps and hamstring	After intervention reduced VL-ST EMG difference was showed during the pre-landing phase for the intervention group

*Only muscle activation study data are summarized.*

*VM, m.vastus medialis; VL, m.vastus lateralis; ST, m.semitendinosus.*

As discussed above the assessment of selected kinematic and kinetic variables during standardized landing and sidecutting maneuvers may be useful to identify gender differences in external loading of the knee joint during sports specific movements. Importantly, in the laboratory setting the magnitude and direction (i.e., frontal, sagittal, and rotational) of external loading can be sensitively quantified, while concurrently counteracted by a host of internal passive (i.e., ligaments and capsule tissue) and active (i.e., musculo-tendinous) forces that in turn can be estimated. From such experiments, the pattern of muscle activation appear to be an important factor to consider when discussing ACL injury risk factors in female athletes (Kaufman et al., 1991).

## Gender Differences in Neuromuscular Activation Patterns (**Table 1**)

Numerous studies have reported that female athletes may systematically demonstrate increased quadriceps activation during landing and cutting maneuvers compared to male athletes (Colby et al., 2000; Malinzak et al., 2001; Zazulak et al., 2005; Sigward and Powers, 2006b; Chappell et al., 2007; Landry et al., 2007; Pollard et al., 2010). Increased quadriceps activation *per se* has been associated with increased anterior tibia shear forces and elevated ACL strain (Sell et al., 2007; Brown et al., 2009; Shultz et al., 2009) and therefore likely is a contributing factor to the elevated risk of non-contact ACL injury observed in female athletes. Further, by means of EMG recordings it has been documented that female athletes tend to have a dominance of lateral quadriceps (VL) activation during side-cutting in contrast to male athletes who tend to demonstrate medial quadriceps (m.vastus medialis) dominance (Myer et al., 2005; Beaulieu et al., 2009). Given that knee abduction is a well-known risk factor for sustaining non-contact ACL injury (Hewett et al., 2005a), elevated activity of the lateral quadriceps would be expected to increase the risk of such injury. However, as discussed in detail above, the hamstrings can play a role as functional ACL synergists, and thus co-activation and/or pre-activation of the medial hamstring muscles (frontal plane antagonist to lateral quadriceps forces) may serve an important stabilizing and protective purpose during landing and rapid side-cutting maneuvers (Ebben et al., 2010; Zebis et al., 2016). When examining the magnitude of agonist-antagonist muscle co-activation during risk movements, gender specific muscle activation (EMG amplitude) patterns characterized by reduced hamstring-to-quadriceps co-activation ratio (H/Q-ratio) have been observed in female athletes compared to male athletes in the pre-touch down phase prior to ground contact when performing sidecutting or vertical drop landings (Nagano et al., 2007; Hanson et al., 2008; Bencke and Zebis, 2011), while also observed in sidecutting during the subsequent contact phase (Hanson et al., 2008; Ebben et al., 2010). In contrast, a single study failed to observe any gender differences in thigh muscle activation when examining college athletes during landing from a jump, which might at least in part be due to a very small sample sizes (8 female vs. 6 male subjects) (Fagenbaum and Darling, 2003).

It remains unclear why female athletes demonstrate a lower H/Q-ratio of muscle activity during risk situations for non-contact ACL injury in sports, where an elevated hamstring activity otherwise would be beneficial for providing dynamic knee joint stability. One potential explanation could be that reduced magnitude of hamstring-quadriceps muscle co-contraction would favor a more explosive jumping movement, as the knee extensors would be able to more efficiently produce high eccentric landing- and elevated concentric push-off force and power. Indeed, high school novice female soccer players demonstrated increased amounts of hamstring-quadriceps co-contraction during sidecutting, which was suggested to reflect an immature pattern of muscular activation (Sigward and Powers, 2006a). In terms of maturation *per se*, children appear to display substantially less muscle co-contraction prior to landing from a jump compared to adults, arguably as a result of using different landing strategies (Russell et al., 2007).

## Neuromuscular Acl Injury Risk Factors Identified by Prospective Designs (**Table 2**)

Only a single prospective study was identified to have examined muscle activity pattern as an isolated risk factor for non-contact ACL injury, in which 55 adult female elite team handball and football players (mean age of 24 years) without previous history of ACL injury were investigated (Zebis et al., 2009). No prospective studies investigating muscle activation deficits and ACL injury risk could be identified in adolescent or young female athletes. In the study by Zebis et al. (2009), it was reported that reduced medial hamstring activity during sidecutting as well as an elevated difference in normalized EMG activity between lateral quadriceps muscle (i.e., m.vastus lateralis) and medial hamstring muscle group [i.e., m.semitendinosus (ST)] were factors that predicted future episodes of non-contact ACL injury (Zebis et al., 2009). As such, these observations underline the importance of medial hamstring muscle activation for providing protection against non-contact ACL injury in female football and team handball athletes.

## Effects of Preventive Exercise Training on Muscle Activation Patterns (**Table 3**)

Specific modes of training have been reported to result in altered neuromuscular coordination patterns during landing, jumping, and sidecutting (Zebis et al., 2008, 2016; Nagano et al., 2011; Letafatkar et al., 2015). Thus, a recent study using postural balance exercises and verbal instructions to land softly reported improved EMG H/Q-ratios prior to and after initial contact in single-leg drop landings in response to a 6-week intervention protocol in female university athletes (Letafatkar et al., 2015). An uncontrolled study in eight young female athletes employed a 5 weeks intervention protocol consisting of plyometric jump and landing exercises that were implemented in selected basketball exercises, which resulted in elevated hamstring muscle activation in the 50 ms time interval prior to landing indicating a more protective recruitment pattern after the period of training (Nagano et al., 2011). Likewise, Zebis et al. (2008) showed that a training program, previously documented to reduce the incidence of non-contact ACL-injury in female team handball players (Myklebust et al., 2003) and consisting of standing balance exercises, landing exercises and game specific jumping exercises, led to increased pre-activity in the medial hamstrings (i.e., m.semitendinosus) during standardized sidecutting maneuvers performed in adult female elite football and handball players (Zebis et al., 2008). Later, the same training protocol was repeated in a group of adolescent female football and team handball players, which resulted in increased medial hamstring-to-lateral quadriceps muscle activation levels compared to matched controls (Zebis et al., 2016). Using another type of intervention protocol, Wilderman et al. (2009) carried out a RCT (*n* = 30) investigating the effect of an agility training program lasting 6 weeks (Wilderman et al., 2009). In accordance with the above studies by Zebis et al. (2008, 2016) and Wilderman et al. (2009) also reported increased medial hamstring activity during sidecutting in young female basketball players, however, the increase was apparent during the contact phase of the sidecutting maneuver and not in the pre activity period immediately prior to contact as reported by the previous studies by Zebis et al. (2008, 2016), Nagano et al. (2011), and Letafatkar et al. (2015). The difference in neuromuscular effect might reflect differences in the specific exercises used, as the agility exercises applied by Wilderman et al. (2009) primarily consisted of exercises emphasizing speed in shuffling the feet and changing directions, while the aforementioned studies focused more on standing balance exercises, balance in landing exercises and joint control during sports-specific exercises.

The above studies suggest that increased medial hamstring muscle activity during high injury risk movements represents an important neuromuscular adaptation to ACL injury prevention training, and that uniform adaptive responses can be achieved in both adult and adolescent female athletes. As discussed in detail above, the protective role of a high medial hamstring activity may be to limit the risk of excessive dynamic valgus and external rotation of the knee joint and thereby reduce stress forces and strain in the ACL. Since no studies so far have performed long term follow-up, it remains unknown for how long the observed adaptation in motor program execution (elevated ST activation) can be sustained in the absence if training or when performing a reduced frequency of training. Also no studies exist on the effect of age or maturation on the sustainability of the improved motor programs.

Most of the included studies investigated young female adults and only few (*n* = 6) comprised adolescent female athletes (age: 10–19 years). Given that the highest risk of non-contact ACL injury appears to exist during the adolescent years (Renstrom et al., 2008) and that patterns of muscular activation have been shown to represent a significant risk factor for ACL injury (Zebis et al., 2009), it seems striking that only a few experimental studies have been performed in this age group. No clear explanation for this lack of studies on muscle activation in adolescent athletes is evident, except that studies using EMG analysis generally are highly time consuming to conduct, and that elaborate efforts of obtaining ethical approval typically are required when studying adolescent athlete populations. However, in the light of the relatively drastic changes in landing biomechanics and lower limb muscle strength as a result of maturation, experiments to study muscle activation patterns in adolescent female athletes seem highly warranted.

## Summary and Perspectives

Collectively, the available data suggests that selected biomechanical risk factors such as anterior shear forces, external knee abduction moments and internal/external knee joint rotation (**Figure 1**) – all factors known to stress the ACL during injury risk situations like landings and sidecutting – may be significantly counteracted by internal joint forces generated by the hamstring muscles. Furthermore, increased hamstring muscle activation and reduced quadriceps activation both would be expected to counteract the magnitude of anterior tibial shear forces, and elevated activation of the medial hamstring muscles (ST in particular) without or in combination with reduced activity in the lateral quadriceps muscle (VL) is expected to counteract external knee abduction (valgus) moments in the frontal plane that are known to represent a strong risk factor for non-contact ACL injury. When landings or cutting maneuvers are performed with extended knee joint angles, also a well-known high risk situation, increased medial hamstring activity would also be expected to protect the knee joint against excessive external rotation moments. Consequently, increased focus on avoiding (or even de-programming) non-optimal patterns of muscle activation seems highly important for reducing the incidence of non-contact ACL injury in young female athletes.

In conclusion, this review shows that young and adult female athletes, in comparison with male athletes, often demonstrate muscle activation patterns during high risk situations in sports that put the athletes at increased risk of sustaining non-contact ACL injury. Specifically, young as well as adult female athletes tend to have higher levels of quadriceps muscle activation, which may contribute to increase the magnitude of anterior tibial shear forces to cause significant ACL strain. In addition (and conjunction), female athletes tend to demonstrate reduced levels of hamstring muscle activation during high-risk movement conditions (sidecutting and stop landing), which further provides less protective capacity for ACL unloading. In the frontal plane, the importance of increased activity in the medial hamstring muscles have been demonstrated, while the balance of medial hamstring muscle activity in relation (proportion) to lateral quadriceps activity appears to particularly influence the magnitude of dynamic stabilization in the knee joint during sports specific high-risk situations. However, considering the fact that adolescent female athletes are at particular high risk of sustaining non-contact ACL injury, there is a striking lack of cross-sectional as well as prospective data on the influence of specific muscle activation patterns on the risk of ACL-injury in this age and gender group. In addition, almost no knowledge exists on the adaptive change in hamstring vs. quadriceps muscle activation patterns during high risk movements evoked by prophylactic neuromuscular exercise training. Consequently, further research is warranted to (1) investigate how certain patterns of lower limb muscle activation may represent a significant risk factor in relation to non-contact ACL injury, and (2) to develop more effective preventive intervention programs targeting adolescent and young female athletes.

## Author Contributions

JB took part in decision on structure and content of the review, performing literature, search, and writing the review. PA and MZ took part in decision on structure and content of the review, contributed to writting the review and gave thorough feedback throughout the process, and accepting the final version.

## Conflict of Interest Statement

The authors declare that the research was conducted in the absence of any commercial or financial relationships that could be construed as a potential conflict of interest.
